# Do individual or societal consequences matter more? Reducing antibiotic expectations for uncomplicated urinary tract infections in females

**DOI:** 10.1111/bjhp.70109

**Published:** 2026-09-06

**Authors:** Elisabeth D. C. Sievert, Sarah Eitze, Cornelia Betsch

**Affiliations:** ^1^ Health Communication, Implementation Science Bernhard Nocht Institute for Tropical Medicine Hamburg Germany; ^2^ Institute for Planetary Health Behaviour University of Erfurt Erfurt Germany

**Keywords:** antibiotic resistance, antibiotic stewardship, patient expectations, risk communication, trust, women's health

## Abstract

**Objectives:**

Antimicrobial resistance (AMR) is an urgent public health threat. Antibiotic prescriptions in primary care, such as for uncomplicated urinary tract infections (UTIs) in females, contribute substantially to antibiotic overuse. We examined whether consequence‐based risk communication emphasizing either individual (gut microbiome disruption) or societal (AMR) consequences can reduce expectations to receive antibiotics and improve decision‐related outcomes.

**Design and Methods:**

Across three online experiments (*N* = 3057), women in the United Kingdom were exposed to a hypothetical physician consultation for an uncomplicated UTI. Depending on the experimental condition, the physician explained individual consequences of antibiotic use, societal consequences or no such consequences. Outcomes included expectations to receive antibiotics, trust in the physician and decisional conflict.

**Results:**

An internal meta‐analysis showed that both societal and individual consequence messages significantly reduced patients' expectations to receive antibiotics. Neither message type undermined trust in the physician (in Studies 1 and 2), and both communication messages significantly reduced decisional conflict (in Study 3).

**Conclusions:**

These findings indicate that brief, targeted communication regarding consequences of antibiotic use can lower antibiotic expectations and support decision‐making without eroding physician trust. Such messages represent a useful tool that can be adapted to different clinical contexts for patient‐centred antibiotic stewardship in primary care.

**Statement of Contribution**

**What is already known on this subject?**

- Patient expectations are an important driver of antibiotic prescribing in primary care, including for uncomplicated urinary tract infections (UTIs) in women, where inappropriate prescribing contributes to antimicrobial resistance (AMR).
- Communicating the societal consequences of antibiotic use, such as AMR, can reduce patients' expectations to receive antibiotics, but evidence comes largely from non‐UTI contexts.
- It remains unclear whether messages emphasizing individual harms, such as gut microbiome disruption, or societal harms, such as AMR, are more effective in reducing expectations in UTI consultations.

**What does this study add?**

- Individual and societal harm messages both reduce expectations for UTI antibiotics.
- These brief consequence messages do not undermine patients' trust in the physician.
- Consequence messages also reduce decisional conflict during consultations.

## INTRODUCTION

Uncomplicated urinary tract infections (UTIs) are among the most common reasons for antibiotic prescriptions in women in primary care (Pujades‐Rodriguez et al., 2019). Although UTIs are often self‐limiting (Ferry et al., 2004), and UK guidelines encourage initial self‐care and delayed prescribing (i.e., the precautionary prescription) and watchful waiting instructions; (NICE, 2018), antibiotics are often prescribed without clear clinical indication (Cox et al., 2023; Ghouri et al., 2020).

Women are disproportionately affected by UTIs and receive a larger share of UTI‐related antibiotic prescriptions (Pujades‐Rodriguez et al., 2019). Patient expectations to receive antibiotics are common and can contribute to prescribing pressure during the clinical encounter. Prior research has conceptualized such expectations as a broader construct (Hamm et al., 1996; Sirota et al., 2017) encompassing both beliefs in antibiotics' effectiveness and expectations towards the physician to prescribe them (Thorpe et al., 2021). They are clinically relevant because they can increase prescribing, both by directly shaping patients' requests or by creating implicit or explicit pressure on clinicians during the consultation (Cole, 2014; McNulty et al., 2013; Sirota et al., 2017) and may be reinforced by underestimating the risks of unnecessary antibiotics use and overestimating their benefits (Cox et al., 2023; Spicer et al., 2020). Patient‐facing risk communication is therefore a relevant route for reducing expectations to receive antibiotics when they may not be clinically warranted.

Consequence communication may be a useful lever because unnecessary antibiotic use has both individual and societal costs: At the individual level, antibiotics can disrupt the gut microbiota, with potential downstream consequences for immune functioning (Langdon et al., 2016). At the societal level, antibiotic overuse contributes to antimicrobial resistance (AMR), thereby undermining treatment effectiveness and increasing morbidity, mortality and health care costs (Laxminarayan et al., 2013; Naghavi et al., 2024). Experimental studies show that communicating the societal consequences of unnecessary antibiotic use (i.e., contributions to AMR) can lower patients' expectations to receive antibiotics for ear infections (Gross et al., 2025; Sievert et al., 2024). At the same time, experimental work on framing suggests that emphasizing individual‐level harms of unnecessary antibiotics can also reduce antibiotic demand and may, in some contexts, be more effective than emphasizing societal harms alone (Miller et al., 2020), while direct comparisons do not always yield robust differences across studies (Stenlund et al., 2024). Thus, it remains an open question whether societal consequence communication (AMR) or individual‐level consequence messaging (e.g., disruption of the gut microbiome) is more effective in reducing patients' expectations to receive antibiotics in the context of uncomplicated UTIs.

This question is theoretically relevant because many established health psychological models, such as the Health Belief Model (HBM; Rosenstock, 1974) or the Theory of Planned Behavior (Ajzen, 1991), focus on individual‐level determinants such as perceived risk, expected benefits or attitudes. While this helps explain why people see immediate symptom relief or view antibiotics as beneficial, these models tend to devote less attention to the societal consequences of individual health behaviour. This matters because individual antibiotic use contributes to the spread of antibiotic‐resistant bacteria (Laxminarayan et al., 2013; Prestinaci et al., 2015), making a seemingly private medical decision a broader societal harm by eroding the future effectiveness of antibiotic treatments. Recent work suggests that such societal consequences can shape health decisions when they are made salient (Böhm & Betsch, 2022). The present research therefore examines whether expectations to receive antibiotics are informed not only by individual‐level considerations like the effects on the gut microbiota, but also by communicated societal consequences such as AMR.

The two consequence types may differ in how they are processed. Societal consequences can evoke altruistic and moral concerns (Betsch et al., 2013, 2017; Böhm et al., 2016; Korn et al., 2020), but tend to be perceived as psychologically distal and abstract, as they relate to future others and outcomes at the population level. Individual consequences seem more proximate and can heighten personal risk perceptions, a core determinant of behaviour in the HBM (Rosenstock, 1974). For women experiencing painful UTI symptoms, immediate symptom relief may outweigh potential distant future risks unless these are communicated in a proximate, tangible and urgent manner (Loewenstein et al., 2001; Trope & Liberman, 2010), making it necessary to empirically compare the effectiveness of both approaches in shaping antibiotic expectations.

Because immediate antibiotics are not always clinically necessary in uncomplicated UTIs (NICE, 2018), efforts to reduce antibiotic expectations are most plausible when embedded in a consultation that offers a management pathway. UK guidance recommends self‐care, including analgesics for pain and a back‐up or immediate antibiotic depending on symptom severity and risk of complications (NICE, 2018). However, analgesics address symptom relief rather than providing a non‐antibiotic substitute for the treatment itself. Plant‐based approaches have been investigated more directly as potential non‐antibiotic or antibiotic‐sparing options in uncomplicated cystitis, although the current evidence remains mixed and further evaluation is needed (Gágyor et al., 2021; Kranz et al., 2024; Moore et al., 2019). Yet, we included a plant‐based treatment option to represent a non‐antibiotic alternative to immediate antibiotic use to examine whether consequence communication can lower antibiotic expectations without implying therapeutic inaction by withholding medication.

We examined these questions in three preregistered online experiments using hypothetical patient scenarios. We tested the impact of individual versus societal consequence communication on women's expectations to receive antibiotics for UTIs as a primary outcome (RQ1) and two secondary outcomes (RQ2)^1^: trust in the physician (Studies 1–2), since expectations must be lowered without undermining the physician–patient relationship, and decisional conflict (Study 3), since highlighting non‐antibiotic options and trade‐offs may either clarify choices or heighten uncertainty.

## STUDY 1

We recruited 581 UK‐based female adults via the online platform Prolific. Sample size was determined by an a priori power analysis (*f* = .15, *α* = .05, 1 − *β* = .95), with 15% oversampling to account for potential exclusions. Participants had to be assigned female at birth and to have experienced fewer than two UTIs in the past 6 months, as chronic UTIs require pathogen‐specific treatment. The study was ethically approved by the University of Erfurt [2024‐11]. Participants were compensated £.88 for completing the six‐minute study.

### Hypotheses

We hypothesized two main effects for consequence communication. Compared to a control condition describing the same consultation context but without any consequence communication, we expected that communicating the individual (H1) or societal (H2) consequences of antibiotic use should both reduce expectations for antibiotics. We additionally explored whether trust in physicians differed across individual (RQ1) and societal (RQ2) consequence communication.

### Design and procedure

We employed a 2 (individual consequences: yes vs. no) × 2 (societal consequences: yes vs. no) between‐subjects design. After providing informed consent, participants were randomly assigned to one of the four conditions and asked to vividly imagine experiencing symptoms consistent with an uncomplicated UTI (lower abdominal pain, frequent and painful urination, feverishness). After symptoms worsened, they were described as visiting a physician who conducted a standard diagnostic assessment (physical exam, urine sample, vital signs). The physician confirmed a mild bladder infection and explained that antibiotics are typically required for more severe infections and that half of mild bladder infections resolve without them. The physician recommended first trying a plant‐based pharmacological treatment, which can also help alleviate symptoms and support recovery. Still, the patient would also receive a precautionary antibiotic prescription. In the individual consequence condition, participants were informed that antibiotic use can disrupt the gut microbiome with negative downstream health effects. In the societal consequence condition, they were told that antibiotic overuse contributes to AMR, which reduces antibiotic effectiveness for others. Finally, expectations to receive antibiotics and trust in the physician were measured.

### Measures

*Expectations to receive antibiotics* were assessed via the mean of six items on a 7‐point Likert scale ranging from 1 (*strongly disagree*) to 7 (*strongly agree*) (Sievert et al., 2024; Thorpe et al., 2021); for example, ‘Antibiotics will relieve my symptoms’ and ‘I would expect that my physician will prescribe antibiotics.’ Higher scores indicated stronger expectations to receive antibiotics (Ω = .96).

*Trust in the physician* was measured using the mean score of the 11‐item Trust in Physician Scale (Anderson & Dedrick, 1990); for example, ‘I trust my physician to put my medical needs above all other considerations when treating my medical problems.’ and ‘My physician is a real expert in taking care of medical problems like mine.’ rated on the same 7‐point scale (Ω = .93).

### Statistical analysis

A two‐way between‐subjects ANOVA was conducted with individual and societal consequence communication as independent variables, and expectations to receive antibiotics as the primary dependent variable. An exploratory ANOVA examined trust in the physician.

### Results

**FIGURE 1:**
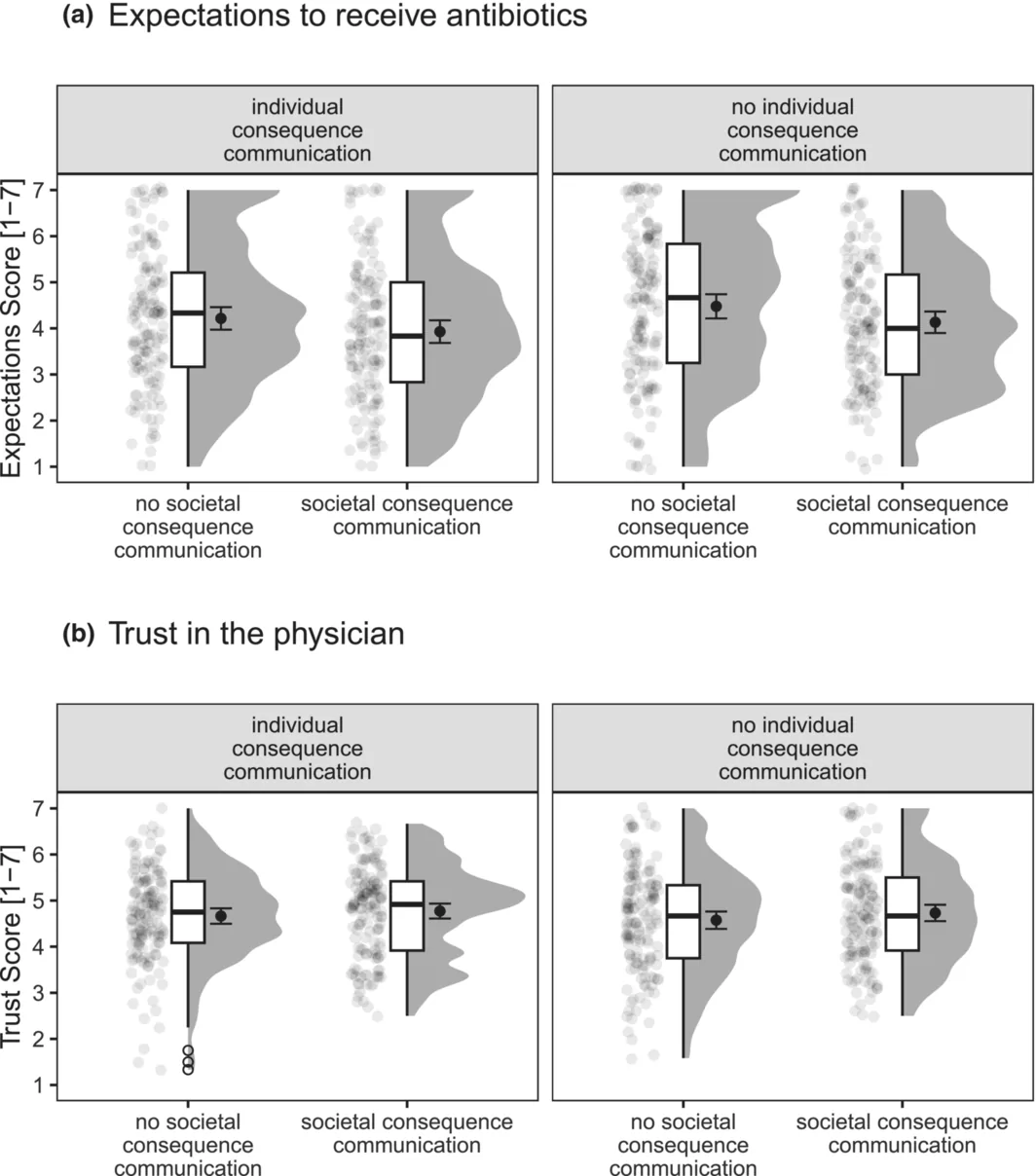
Distributions and means (95% CIs) for expectations and trust. Panel a displays antibiotic expectations, Panel b displays trust in the physician. Both show the means and 95% Confidence Intervals (CI), boxplots and raincloud plots for distributions. Higher scores indicate higher expectations (a), and higher trust (b). Societal consequence communication significantly lowered expectations to receive antibiotics. All other differences between the experimental groups on expectations or trust are non‐significant.

As displayed in Figure 1a, individual consequence communication (H1) regarding disruption of the gut microbiome had no significant effect on expectations to receive antibiotics, *F*(1, 577) = 3.53, *p* = .061, *η* ^2^ = .006. In contrast, highlighting the societal risks of antibiotic overuse (H2), i.e., contributing to AMR, significantly reduced participants' expectations to receive antibiotics, *F*(1, 577) = 6.41, *p* = .012, *η* ^2^ = .011. The interaction between individual and societal consequences was not significant, *F*(1, 577) = .06, *p* = .814, *η* ^2^ < .001.

Neither the communication of individual consequences (RQ1), *F*(1, 557) = .56, *p* = .455, *η* ^2^ = .001, nor the communication of societal consequences (RQ2), *F*(1, 557) = 2.27, *p* = .133, *η* ^2^ = .004 had an impact on trust in the physician. The interaction was also non‐significant, *F*(1, 557) = .08, *p* = .782, *η* ^2^ < .001 (see Figure 1b). To investigate whether the null findings reflect negligible effects on trust, we conducted exploratory two one‐sided tests for equivalence (TOST) (Lakens et al., 2018), setting the bounds to *d* = ±.20, the smallest effect of interest according to Lakens (2013), see Supplement, Table S4. Equivalence was established for the individual versus control contrast (effects < *d* = .20). For the societal versus control contrast, equivalence was not established; thus, the evidence remains inconclusive whether very small negative effects on trust can be ruled out.

### Discussion

Study 1 replicated prior findings that societal consequence communication can lower expectations to receive antibiotics (Gross et al., 2025; Sievert et al., 2024), whereas the individual consequence communication on gut microbiome disruption showed no effect. One possible explanation is that the individual message may have been too abstract or technical. Thus, in Study 2, we tested whether a more accessible communication of individual consequences would be more effective.

The evidence from the ANOVA and TOST analyses regarding the effect of the two communication strategies on trust in the physician was inconclusive, indicating that further research is needed before drawing conclusions regarding the physician–patient relationship.

The societal consequence message had the expected effect on expectations. Societal consequences are unevenly distributed: Women bear a disproportionate burden of antibiotic need and AMR‐related treatment failure (Batheja et al., 2025; Lynch et al., 2024), reflecting heightened antibiotic requirements due to pregnancy, childbirth or post‐abortion care, alongside increased UTI susceptibility due to anatomy, poor sanitation or sexual violence (Minardi et al., 2011). In our female participants, making these distributional consequences salient versus not salient may increase the impact of the societal message (Batson, 2011; Fehr & Schmidt, 1999; Schwartz, 1977). Highlighting relevant potential harms may heighten self‐relevance, identification and concern, thereby increasing the subjective weight of social costs in decision‐making (Tajfel & Turner, 1979). This framing may reduce psychological distance and therefore elicit stronger responses than a generic population‐level framing (Trope & Liberman, 2010). Emphasizing existing inequalities and injustice may, however, also carry relational risks if perceived as moralizing, which could impede trust in the physician communicating the message (Elwyn et al., 2012; Hall et al., 2001).

To address the potential limitations of the individual message and test whether adding an inequality explanation makes the societal message even more effective and/or compromises trust, we conducted a formative pretest followed by a second study to replicate the experiment with revised materials.

## STUDY 2

A pretest with *n* = 30 women from the target population evaluated the clarity and accessibility of both consequence messages. Feedback from open‐ended questions indicated that the original individual consequence message regarding gut microbiome disruption was indeed perceived as too technical and abstract; we therefore simplified the wording for better comprehensibility without altering the core content.

### Hypotheses

We hypothesized that when participants receive a description of a typical consultation situation participants would report lower expectations for antibiotics when individual (H1) or any type of societal consequences (H2) were communicated, compared to when the situation was described without any reference to potential consequences (main effects of individual and societal consequences, respectively). We also explored whether adding the inequality information changed the effect of societal consequence communication on expectations and trust.

### Method

An a priori power analysis (f = .15, α = .05, 1 − β = .95) determined the required sample size, with 20% oversampling to account for exclusions given the higher‐than‐expected prevalence of individuals with >1 UTI in the past 6 months in Study 1. We recruited 765 UK‐based female participants via Prolific, who were compensated £.70 for completing the 7‐min study. The study was ethically approved by the University of Erfurt [2024‐36].

Participants were randomly assigned to a 2 (individual consequences: yes vs. no) × 3 (societal consequences: AMR vs. AMR + inequalities vs. none) between‐subjects design. We described the same hypothetical scenario as in Study 1 with a slightly simplified wording as a result of the pretest. In the new AMR + inequalities condition, participants were informed that women are at higher risk of experiencing future AMR‐related burden throughout their lives due to greater antibiotic need in pregnancy, childbirth and anatomical susceptibility to infections, thereby explaining AMR as a gendered distributional harm. This enabled testing whether adding a gender‐based inequity frame explicitly emphasizing women's higher vulnerability to the future societal consequences would enhance the persuasive impact compared to the societal‐consequence message alone. *Expectations to receive antibiotics* and *trust in the physician* were measured as in Study 1.

### Statistical analysis

We conducted a 2 × 3 between‐subjects ANOVA with individual and societal consequence communication as independent variables, and expectations to receive antibiotics as the primary dependent variable to test for main and interaction effects, complemented by exploratory Tukey‐adjusted post‐hoc pairwise comparisons. An exploratory ANOVA examined trust in the physician.

### Results

**FIGURE 2:**
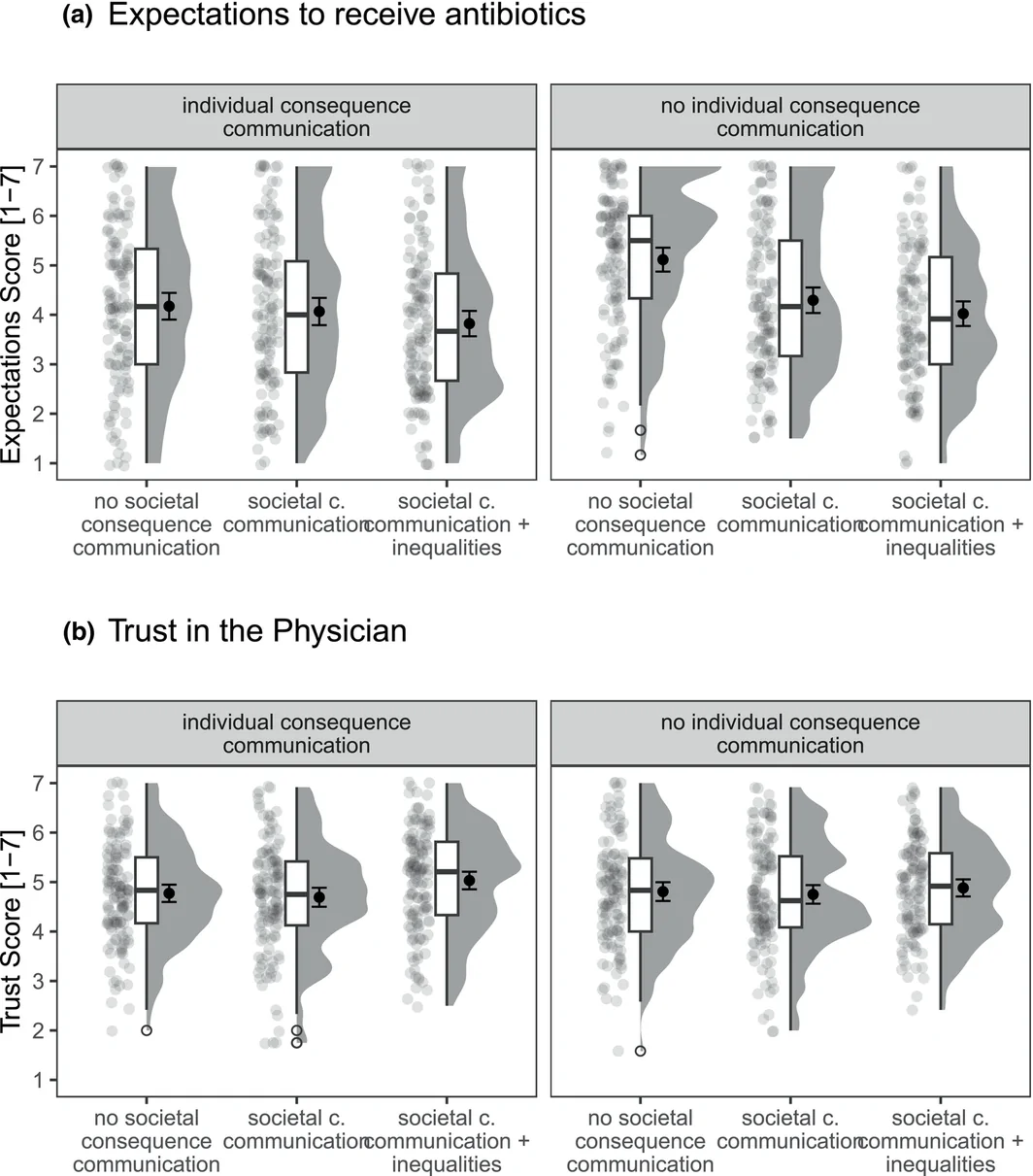
Distributions and means (95% CIs) for expectations and trust. Panel a displays antibiotic expectations; Panel b displays trust in the physician. Both show the means and 95% Confidence Intervals (CI), boxplots and raincloud plots for distributions. Higher scores indicate higher expectations (a) and higher trust (b). Individual and societal consequence communication significantly lowered expectations to receive antibiotics.

Figure 2a depicts that participants exposed to individual consequence communication reported lower expectations, *M* = 3.95, 95% CI [3.80; 4.10], than those not exposed to individual consequence communication, *M* = 4.49, 95% CI [4.34, 4.64], *F*(1, 759) = 24.89, *p* < .001, *η* ^2^ = .03, in line with H1. Similarly, expectations differed by societal consequence communication, *F*(2, 759) = 14.71, *p* < .001, *η* ^2^ = .04, in line with H2, with highest expectations when no societal consequences were communicated, *M* = 4.63, 95% CI [4.45; 4.81], compared with AMR communication, *M* = 4.09, 95% CI [3.91, 4.27] and AMR + inequalities communication, *M* = 3.94, 95% CI [3.76, 4.12]. The two societal consequence conditions did not differ, *M*diff = .15, 95% CI [−.16, .46], *p* = .490. The significant interaction, *F*(2, 759) = 4.76, *p* = .009, *η* ^2^ = .01, suggests that providing societal consequence information had a stronger effect when there was no information on the individual consequences present, *M*diff = 1.02, *p* < .001.

Communicating societal consequences had a small influence on trust in the physician, as depicted in Figure 2b, *F*(2, 759) = 4.61, *p* = .010, *η* ^2^ = .01. Trust did not differ significantly depending on the communication of any social or individual consequences (Tukey‐adjusted pairwise comparisons across the six experimental conditions were not significant, *ps* ≥ .103). Neither the main effects nor the interaction was significant. Equivalence tests (Table S6) showed that trust scores in the individual consequence condition were equivalent to control. Both societal messages were not equivalent to the control group, but they were equivalent to each other (societal consequences + inequalities: *M* = 5.00, 95% CI [4.87, 5.13], societal consequence communication without inequalities: *M* = 4.73, 95% CI [4.61, 4.86]; no societal consequence communication: *M* = 4.78, 95% CI [4.65, 4.91]); small positive effects thus cannot be ruled out.

### Discussion

Findings from Studies 1 and 2 showed that societal‐consequence messages consistently reduced expectations to receive antibiotics. However, additionally emphasizing future gender inequalities did not produce an additional increase of the effect. Although the inequality framing is theoretically relevant because it highlights differential vulnerability and broader societal implications, future research is needed to determine whether and in which target groups and consultation contexts it may help reduce antibiotic expectations. Therefore, future studies should integrate sex‐related drivers of antibiotic need, such as UTI susceptibility, with a broader argument that AMR can also exacerbate gender‐related inequalities through structural differences in exposure, access to care and treatment burden across the life course (Batheja et al., 2025).

In Study 2 and in contrast to Study 1, the individual‐consequence message reduced expectations. We assume that this difference to Study 1 occurred because we simplified the explanation of the individual consequences. We further cautiously conclude that consequence communication has no meaningful effects on trust in the physician, at least no negative effects. This is consistent with patterns previously reported by Sievert et al. (2024), where communicating the societal consequences of antibiotic overuse also reduced expectations to receive antibiotics without adversely affecting trust.

In Studies 1 and 2, the physician in the vignette consistently recommended a plant‐based alternative alongside antibiotics. Presenting two potential treatments may have inadvertently increased decisional conflict (O'Connor, 1995), defined as subjective uncertainty about the best course of action, as larger choice sets can make decisions more difficult (Iyengar & Lepper, 2000; Tversky & Shafir, 1992). The plant‐based option was included to reflect a consultation in which expectations to receive antibiotics are lowered without implying therapeutic inaction, by offering a non‐antibiotic treatment alternative to immediate antibiotic use. Although it was intended as a substitute rather than an add‐on, its co‐presentation with a precautionary antibiotic prescription may have created ambiguity. This could have heightened uncertainty about the appropriateness of immediate antibiotic use (Han et al., 2011; Politi et al., 2013) or biased some participants towards precautionary antibiotic use (Cabral et al., 2015; Horwood et al., 2016). The joint recommendation of two treatments may thus have introduced a decision‐structural confound, raising the possibility that observed effects partly reflect differences in perceived decision difficulty rather than differences in consequence communication alone. Study 3 was designed to address this concern.

## STUDY 3

Study 3 had two objectives: To test consequence communication's effects on antibiotic expectations under different physician recommendation conditions (antibiotics vs. antibiotics preceded by a plant‐based alternative), and to examine whether consequence communication also reduces decisional conflict—the perceived uncertainty in treatment decisions involving trade‐offs (O'Connor, 1995). Decisional conflict is theoretically and practically relevant in medical decision‐making (O'Connor, 1995; O'Connor et al., 1999), as it is sensitive to the quality of risk communication and decision support. It tends to decrease when options and their consequences are clearly explained (Stacey et al., 2017), making decisional conflict a relevant evaluation criterion for decision support (Garvelink et al., 2019).

Communicating the consequences of unnecessary antibiotic use while presenting both treatment options may therefore reduce decisional conflict at the point of choice. Making trade‐offs explicit (e.g., rapid symptom relief vs. risks such as adverse effects and AMR) enables alignment with patient values. Without such information, individuals may overweight the immediate effect of antibiotics and default to the more ‘active’ option, consistent with action bias (Patt & Zeckhauser, 2000). From an expectancy–value perspective, consequence information should lower the perceived utility of immediate antibiotics by making associated costs salient (Eccles & Wigfield, 2002; Wigfield & Eccles, 2002). When a clinically acceptable non‐antibiotic substitute is available without these costs, its relative value increases, which should reduce conflict.

Study 3 orthogonally manipulated consequence communication (individual vs. societal vs. control) and treatment recommendation (antibiotic only vs. antibiotic plus alternative) and included decisional conflict as an additional outcome.

### Hypotheses

Again, we hypothesized that both individual (H1a) and societal (H1b) consequence communication would reduce expectations to receive antibiotics compared to a control condition within the same consultation context where no consequences are communicated. We additionally hypothesized that decisional conflict would be greater when the physician communicated negative consequences of antibiotic use but recommended antibiotics as the only option (H2).

### Method

We recruited 1711 UK‐based female participants via Prolific. An a priori power analysis for a 2 × 3 ANOVA including main effects and interactions determined the target sample size (*f* = .15, α = .05, power = .80); we quadrupled this base N by four for adequate interaction power (Giner‐Sorolla et al., 2024) and added 20% for post‐hoc exclusions.

Participants were randomly assigned to a 3 (communication condition: individual vs. societal vs. control) × 2 (treatment recommendation: antibiotics only vs. antibiotics + alternative) between‐subjects design and received £.75 for completing the four‐minute study. Ethical approval was obtained from the University of Erfurt [2025‐09].

### Measures and procedure

*Expectations to receive antibiotics* were measured as in Studies 1 and 2.

*Decisional conflict* was assessed via the mean of the 16‐item Decisional Conflict Scale (O'Connor, 1995), which captures perceived clarity, knowledge and confidence in decision‐making (e.g., ‘I feel I have made an informed choice’), rated from 1 (*strongly disagree*) to 5 (*strongly agree*); higher scores indicate higher conflict (Ω = .98).

Participants were presented with a vignette describing a hypothetical physician's consultation for an uncomplicated UTI. Depending on the experimental condition, the physician either communicated individual consequences of antibiotic use (disruption of the gut microbiome), societal consequences (contribution to AMR) or neither. Subsequently, expectations to receive antibiotics were measured. Afterwards, depending on condition, the physician either recommended initiating antibiotics if symptoms worsened, or they recommended trying a plant‐based pharmacological treatment first while providing a precautionary antibiotic prescription. We then measured participants' decisional conflict.

### Statistical analysis

The preregistration had erroneously specified a 2 × 3 between‐participants ANOVA for expectations, even though the second experimental factor (treatment recommendation) was introduced only after expectations had been assessed and could not have affected expectations. Expectations to receive antibiotics were therefore analysed using a one‐way ANOVA with consequence communication as the only factor. Decisional conflict was analysed with the preregistered 3 × 2 ANOVA, examining the effects of consequence communication and treatment recommendation.

### Results

**FIGURE 3:**
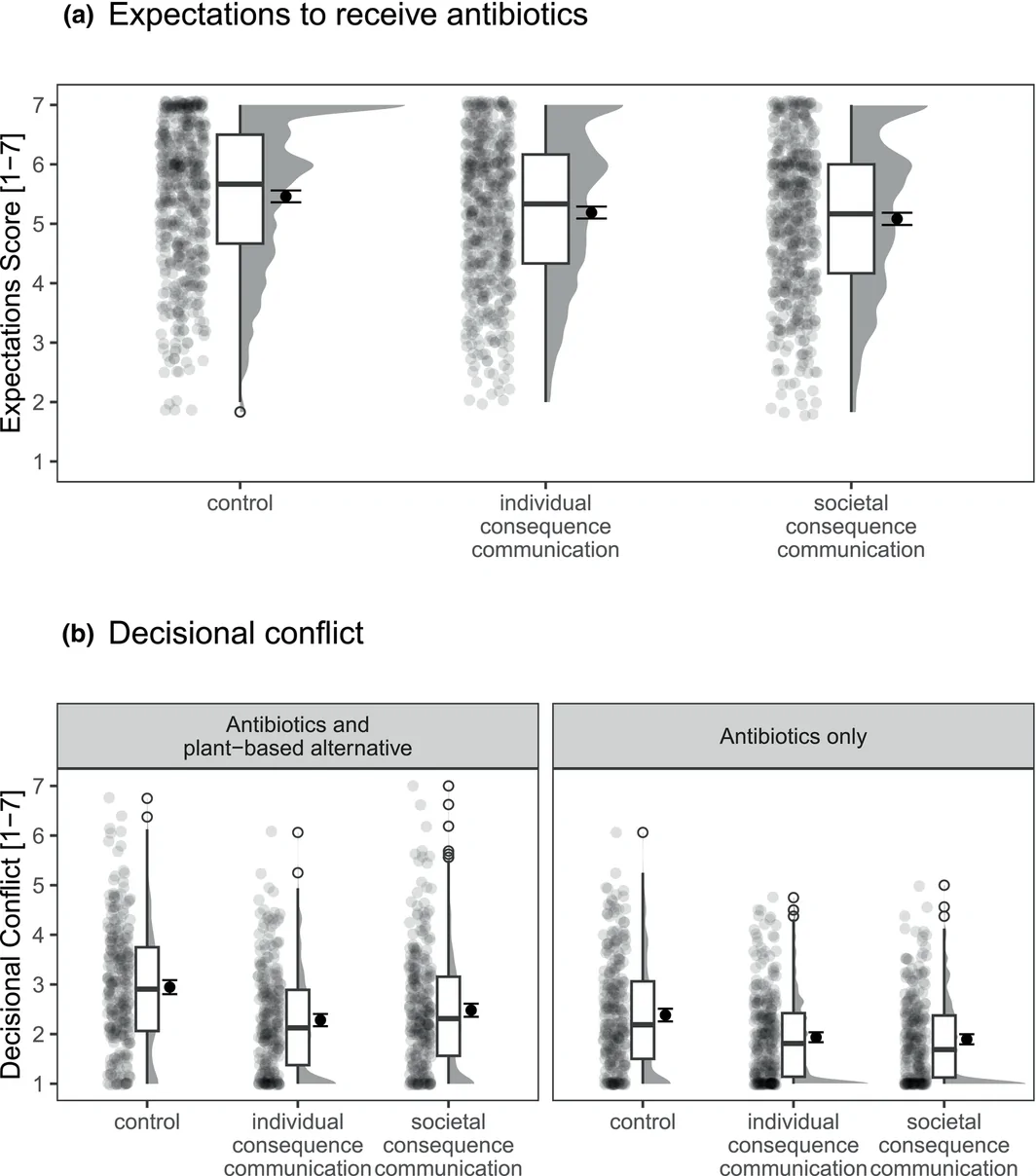
Distributions and means (95% CIs) for expectations and decisional conflict. Panel a displays antibiotic expectations, Panel b displays decisional conflict. Both show the means and 95% Confidence Intervals (CI), boxplots and raincloud plots for distributions. Higher scores indicate higher expectations (a) and higher decisional conflict (b). Individual and societal consequence communication significantly lowered expectations to receive antibiotics.

A one‐way ANOVA revealed the significant effect of consequence communication type on participants' expectations to receive antibiotics that was expected in H1, *F*(2, 1708) = 14.31, *p* < .001, *η* ^2^ = .02. Figure 3a and post‐hoc comparisons indicated that compared to the control condition (*M* = 5.46, 95% CI [5.36; 5.56]), participants in both the individual condition (*M* = 5.19, 95% CI [5.09; 5.29], *p* < .001, *d* = .22) and the societal condition (*M* = 5.08, 95% CI [4.98; 5.19], *p* < .001, *d* = .31) reported significantly lower expectations. The difference between the individual and societal conditions was not significant, *M*diff = .11, 95% CI [−.07, .28], *p* = .315, *d* = .09.

For decisional conflict, Figure 3b shows a main effect of recommendation in that participants who received an additional plant‐based treatment recommendation reported higher decisional conflict, *M* = 2.57, 95% CI [2.50, 2.64], than participants in the antibiotic‐only condition, *M* = 2.07, 95% CI [2.00, 2.14], *F*(1, 1705) = 96.57, *p* < .001, *η* ^2^ = .05. Additionally, there was a main effect of communicating consequences on decisional conflict, *F*(2, 1705) = 47.13, *p* < .001, *η* ^2^ = .05. Decisional conflict was lower in the individual consequence condition, *M* = 2.11, 95% CI [2.02, 2.20] and the societal consequence condition, *M* = 2.19, 95% CI [2.10, 2.27], than in the control condition, *M* = 2.66, 95% CI [2.58, 2.75]. Contrary to H2 assuming that consequence communication would especially reduce conflict when two options are offered, the interaction was not significant, *F*(2, 1705) = 2.27, *p* = .104, *η* ^2^ = .003. As displayed in Figure 3b, participants who received a recommendation for a plant‐based alternative along with their precautionary prescription of antibiotics reported highest decisional conflict overall, particularly when no information about individual or societal consequences of antibiotics was provided (control condition), *M* = 2.95, 95% CI [2.82, 3.07]. Taken together, clear consequence messaging appears to mitigate, but not eliminate the additional decisional burden introduced by presenting a non‐antibiotic alternative.

### Discussion

Study 3 replicated the pattern of Studies 1 and 2 that societal consequence communication is effective in reducing expectations to receive antibiotics. Similarly, communicating the individual consequences also reduced expectations to receive antibiotics, in line with Study 2. These findings suggest that even brief, well‐designed messages can recalibrate expectations—which is important as expectations causally shape physicians' prescribing behaviour (Sirota et al., 2017). This study further reveals that decisions in this context are complex. Adding a non‐antibiotic option to the recommendation of an antibiotic increased decisional conflict, whereas societal or individual consequences of antibiotic use lowered decisional conflict irrespective of the medication recommendation. We had expected that pairing consequence communication with an antibiotic‐only treatment recommendation would heighten decisional conflict due to a mismatch between the consequence communication and the absence of alternative treatment options, but the results did not support this. Given the increase in decisional conflict when a non‐antibiotic alternative was offered, providing clear instructions about which option should be tried first seems to be important.

## COMPARATIVE ANALYSIS ACROSS STUDIES

### Internal meta‐analysis

**FIGURE 4:**
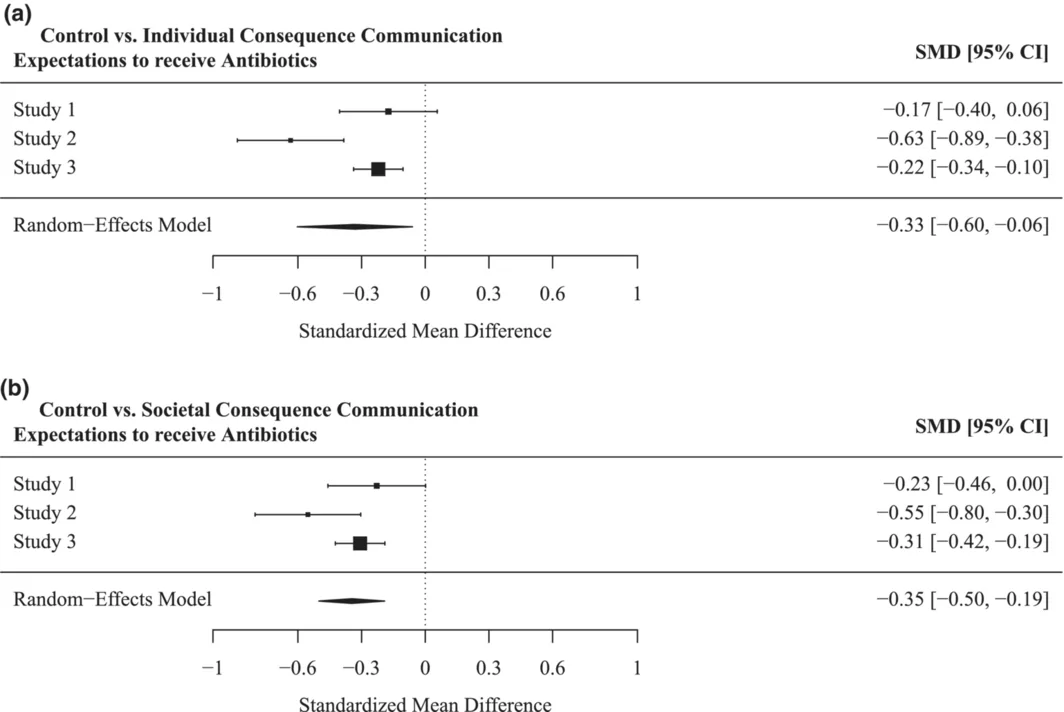
Consequence communication reduces expectations to receive antibiotics (random‐effects meta‐analysis, REML). Panel a: Control versus Individual Consequence Communication (Studies 1–3). Panel b: Control versus Societal Consequences Communication (Studies 1–3). Points show study‐level standardized mean differences (SMD; Hedges *g*) with 95% CIs; diamonds show the REML random‐effects pooled estimates. Negative values indicate lower expectations in the treatment versus control group.

We conducted a meta‐analysis of the structurally comparable conditions across all studies to summarize the results of consequence communication on participants' expectations to receive antibiotics. Using standardized mean differences (SMD; Borenstein et al., 2009), we compared individual consequence communication (vs. control) (Figure 4a) and societal consequence communication (vs. control) (Figure 4b). A random‐effects model with Restricted Maximum Likelihood (REML) estimated between‐study variance.

Figure 4 shows that, except for the comparison involving individual consequences in Study 1, all pairwise comparisons yielded statistically significant results. Overall, both individual (SMD = −.33 [−.60, −.06]) and societal (SMD = −.35 [−.50, −.19]) consequence communication reduced expectations to receive antibiotics compared to the control group without consequence communication.

Overall, the meta‐analytic results reveal small‐to‐medium effect sizes for the reduction in expectations to receive antibiotics after societal or individual consequences relative to the same consultation context without consequence communication. With moderate heterogeneity (I^2^ = 48.74%), the results for societal consequence communication appear to be more consistent across studies than in the individual consequence condition (I^2^ = 82.6%). The high heterogeneity in the individual analysis emphasizes the importance of considering contextual or methodological moderators. Notably, we changed the wording from Study 1 to Study 2 following a pretest. Together, this suggests that societal consequence communication may be more robust in shaping patient expectations. This conclusion should, however, be interpreted cautiously, as the analysis is based on only three closely related studies in a fictitious setting and the more heterogeneous individual‐consequence effects may also reflect methodological differences.

## GENERAL DISCUSSION

Antibiotic overuse in primary care is a major contributor to AMR (Costelloe et al., 2010; O'Neill, 2016), with uncomplicated UTIs in women being a common indication in outpatient prescriptions (Hooton, 2012). This research examined how consequence communication, namely messages highlighting either individual risks (e.g., microbiome disruption) or societal risks (e.g., AMR) of unnecessary antibiotic use, can reduce patients' expectations for antibiotics (RQ1) and affect secondary outcomes of informed and socially responsible decision‐making, such as trust in physicians or decisional conflict (RQ2).

Across three experiments involving a combined sample of over 3000 UK‐based women, both individual and societal consequence communication were associated with lower expectations to receive antibiotics for a hypothetical UTI. This matters because women often expect antibiotics for UTIs but are also open to alternative treatment strategies when adequately informed (Gbinigie et al., 2022; Leydon et al., 2010). Our results replicate earlier findings that societal consequence communication reduces antibiotic expectations (Gross et al., 2025; Sievert et al., 2024) and extend them to the specific clinical context of UTIs, a setting with persistently high rates of unnecessary prescribing and entrenched patient expectations to receive antibiotic treatment. An internal meta‐analysis across the three studies supported this pattern, although effects were small to moderate and more heterogeneous for individual‐consequence messages. Effect sizes of this magnitude are common in health psychology (Webb & Sheeran, 2006) and may still be practically relevant when small shifts at the individual level accumulate into meaningful effects at the population level (Rose, 1985; West, 2007).

The present findings suggest that individual‐consequence messages can reduce antibiotic expectations when they are communicated in a simplified and concrete manner. This aligns with health behaviour theories such as the Health Belief Model (Rosenstock, 1974) which emphasize perceived personal risk as a motivator of behaviour change. At the same time, the effects of societal consequence messaging suggest that antibiotic‐related judgements may not only be influenced by anticipated individual risks, but also by the communication of the broader societal consequences, which are often overlooked in traditional health models (Böhm & Betsch, 2022). Although the present studies were not designed as formal tests of health behaviour theories, they point to a conceptual limitation of models that emphasize individual‐level determinants and make collective aspects of health‐related decision‐making less explicit.

The individual and societal messages represent two communication strategies rather than a single manipulated feature. The aim was to compare these strategies, not to isolate the effect of consequence target while holding all other message characteristics constant. The two messages likely differed in several respects, for instance, in the temporal distance, concreteness or salience of the described consequences. The findings show how the two strategies compare in reducing antibiotic expectations, but they do not identify which specific message features produced the effects.

Importantly, the studies suggest that trust in the physician is largely unaffected by the communication of consequences (Studies 1 and 2) and consequence communication lowered decisional conflict (Study 3). This suggests that consequence communication may lower antibiotic expectations without necessarily impairing patients' experience of the consultation.

### Limitations and future directions

The Trust in Physician Scale (Anderson & Dedrick, 1990) was originally developed in the United States. In our study, however, participants were instructed to answer the items about the physician and consultation shown in the vignette, which matches the single consultation focus of our manipulation. We therefore interpret the trust findings as responses to the depicted consultation rather than to an assumed ongoing relationship.

A further set of limitations is methodological. The hypothetical nature of vignette studies limits ecological validity (Evans et al., 2015), because participants were not experiencing actual symptoms, which may reduce emotional involvement and the salience of decision‐making consequences. Even so, vignettes are an established method of studying judgements and decision‐making in health care, because they allow potential drivers of perceptions and responses to be systematically varied under controlled conditions and thus serve as an efficient early‐stage test bed before piloting interventions in clinical settings (Evans et al., 2015; Sheringham et al., 2021). Additionally, the control condition was not a neutral no‐information control, but the same consultation context without explicit consequence information. This makes the comparison more clinically plausible and conservative but means the reported effects should be interpreted relative to consultations without explicit consequence information rather than a completely neutral control condition. Another limitation concerns the fact that all vignettes included a plant‐based alternative. While intended to reflect a plausible non‐antibiotic treatment option, its positive framing may have shaped participants' responses by providing a substitute to immediate antibiotic use, making people less likely to expect antibiotics. Yet, Study 3 partly addressed this concern as it measured expectations before the recommendation was introduced. Future research should still examine whether similar effects emerge when other non‐antibiotic options are offered or in consultations without an alternative treatment.

Furthermore, our sample included women with and without prior UTI experience. Future research should examine whether such prior experience moderates the effects of consequence communication, as illness and treatment history may shape how firmly antibiotic expectations are held. Beyond that, the central test is whether the effects observed here translate into behaviour—examining consequence communication in real‐world settings such as primary‐care waiting rooms would allow downstream outcomes to be assessed, including prescription fill rates, consultation satisfaction and actual antibiotic use. A final question concerns breadth of application: whether these effects generalize to other conditions in which inappropriate prescribing is common, and across genders, cultures and clinical contexts.

The findings may be particularly relevant in the United Kingdom, where access pathways for uncomplicated UTIs have widened through pharmacy‐based assessment and supply routes (NHS England, 2024a, 2024b). In such settings, it is especially important that patients are aware of the full range of consequences associated with antibiotic use and are supported in making informed decisions. Brief decision aids improve knowledge and can reduce unnecessary antibiotic use (Coxeter et al., 2015; Stacey et al., 2017). For UTIs, guideline‐linked tools and UTI‐specific leaflets can provide symptom‐severity and safety‐netting cues (Lecky et al., 2020; NICE, 2021). Pairing our messages with a simple, UTI‐focused decision aid explaining the differences between mild and severe symptoms and when to seek care could contribute to reducing unnecessary antibiotic use.

## CONCLUSION

Overall, this research suggests that brief, tailored consequence communication—whether emphasizing personal health risks or societal harm—can reduce antibiotic expectations and reduce decisional conflict in females with uncomplicated UTI symptoms. Although future research is needed to investigate whether these effects translate into real‐world prescribing decisions and antibiotic use, the present findings highlight the potential of consequence information as a simple and promising tool for informed decision‐making and antimicrobial stewardship in primary care.

## AUTHOR CONTRIBUTIONS

**Elisabeth D. C. Sievert:** Conceptualization; methodology; formal analysis; data curation; writing – original draft; writing – review and editing; visualization; project administration. **Sarah Eitze:** Conceptualization; methodology; formal analysis; writing – review and editing. **Cornelia Betsch:** Conceptualization; methodology; writing – review and editing; supervision; funding acquisition.

## FUNDING INFORMATION

This research was supported by grants from the German Research Foundation (DFG, grant number: BE 3970/8‐2) and the Leibniz Foundation (Leibniz Best Minds Program 106/2020). The funding source neither influenced the design of the study nor the analysis of the results.

## Supporting information


**Table S1.** Overview of participant characteristics by treatment condition.
**Table S2.** Standardized factor loadings for the one‐factor solution of the expectation items.
**Table S3.** Standardized pattern matrix for the two‐factor solution of expectation and intention items.
**Table S4.** Equivalence tests on trust.
**Table S5.** Overview of participant characteristics by individual conditions.
**Table S6.** Equivalence tests on trust.
**Table S7.** Logistic regression predicting antibiotic intake.
**Table S8.** Logistic regression predicting intake of plant‐based alternative.
**Table S9.** Overview of participant characteristics by recommendation conditions.
**Table S10.** Logistic regression predicting buying antibiotics (no = 0, yes = 1): Odds ratios (Wald 95% CI).
**Table S11.** Logistic regression predicting taking antibiotics (no = 0, yes = 1): Odds ratios (Wald 95% CI).
**Table S12.** Logistic regression predicting buying plant‐based alternative (no = 0, yes = 1): Odds ratios (Wald 95% CI).
**Table S13.** Logistic regression predicting taking plant‐based alternative (no = 0, yes = 1): Odds ratios (Wald 95% CI).

## Data Availability

All anonymized data, analysis code, materials and study documents are available on the Open Science Framework: [https://osf.io/f6wes]. All studies were preregistered: Study 1 (https://aspredicted.org/nznh‐jrrd.pdf), Study 2 (https://aspredicted.org/kyjg‐p6cg.pdf), Study 3 (https://aspredicted.org/8q2n‐2pk9.pdf).

## Footnotes

^1^
Intentions to take antibiotics were preregistered as outcome in Study 1 and additionally measured in the remaining studies. An exploratory factor analysis on Study 1 data indicated that intention and expectation items did not form empirically distinguishable factors. We therefore focus on expectations to receive antibiotics as the primary outcome across all three studies and report the preregistered intention analyses, the factor analysis and measurement details in the Supplementary Materials. As intention items differed in wording and number across studies, we did not conduct a direct cross‐study comparison.
