# Supplementary material for: Do individual or societal consequences matter more? Reducing antibiotic expectations for uncomplicated urinary tract infections in females
Source: Br J Health Psychol. 2026 Sep 6;31(3):e70109. doi: 10.1111/bjhp.70109 (PMC13547770; doi:10.1111/bjhp.70109)
Supplement: Supplementary file 1 [file BJHP-31-0-s001.docx]

**Supplement**

**Do individual or societal consequences matter more? Reducing antibiotic expectations for uncomplicated urinary tract infections in females**

**Study 1**

**Table S1**

| *Overview of participant characteristics by treatment condition* | | | | | |
| --- | --- | --- | --- | --- | --- |
|  | **individual (N=144)** | **societal (N=145)** | **combination (N=145)** | **control (N=147)** | **Overall (N=581)** |
| **Age mean** |  |  |  |  |  |
| Mean (SD) | 42.2 (12.6) | 39.9 (13.5) | 40.8 (12.3) | 40.2 (12.8) | 40.8 (12.8) |
| Median [Min, Max] | 40.5 [21.0, 73.0] | 37.0 [21.0, 74.0] | 38.0 [20.0, 82.0] | 38.0 [18.0, 75.0] | 38.0 [18.0, 82.0] |
| **Age distribution** |  |  |  |  |  |
| 18-30 | 27 (18.8%) | 44 (30.3%) | 36 (24.8%) | 35 (23.8%) | 142 (24.4%) |
| 31-40 | 45 (31.3%) | 42 (29.0%) | 45 (31.0%) | 51 (34.7%) | 183 (31.5%) |
| 41-50 | 34 (23.6%) | 26 (17.9%) | 30 (20.7%) | 31 (21.1%) | 121 (20.8%) |
| 51-60 | 23 (16.0%) | 18 (12.4%) | 20 (13.8%) | 18 (12.2%) | 79 (13.6%) |
| 61-70 | 13 (9.0%) | 12 (8.3%) | 13 (9.0%) | 10 (6.8%) | 48 (8.3%) |
| 71-80 | 2 (1.4%) | 3 (2.1%) | 0 (0%) | 2 (1.4%) | 7 (1.2%) |
| 81-94 | 0 (0%) | 0 (0%) | 1 (0.7%) | 0 (0%) | 1 (0.2%) |
| **Education** |  |  |  |  |  |
| no school | 0 (0%) | 0 (0%) | 0 (0%) | 0 (0%) | 0 (0%) |
| some high school | 2 (1.4%) | 2 (1.4%) | 1 (0.7%) | 3 (2.0%) | 8 (1.4%) |
| high school diploma or equivalent | 19 (13.2%) | 21 (14.5%) | 20 (13.8%) | 29 (19.7%) | 89 (15.3%) |
| some college but no degree | 15 (10.4%) | 14 (9.7%) | 20 (13.8%) | 10 (6.8%) | 59 (10.2%) |
| Trade/technical/vocational training | 15 (10.4%) | 8 (5.5%) | 14 (9.7%) | 6 (4.1%) | 43 (7.4%) |
| associate's degree | 2 (1.4%) | 6 (4.1%) | 2 (1.4%) | 3 (2.0%) | 13 (2.2%) |
| bachelor's degree | 61 (42.4%) | 64 (44.1%) | 55 (37.9%) | 61 (41.5%) | 241 (41.5%) |
| master's degree | 21 (14.6%) | 21 (14.5%) | 20 (13.8%) | 22 (15.0%) | 84 (14.5%) |
| Professional degree | 2 (1.4%) | 4 (2.8%) | 5 (3.4%) | 9 (6.1%) | 20 (3.4%) |
| doctorate | 6 (4.2%) | 5 (3.4%) | 6 (4.1%) | 3 (2.0%) | 20 (3.4%) |
| other | 1 (0.7%) | 0 (0%) | 2 (1.4%) | 1 (0.7%) | 4 (0.7%) |
| **Experience with UTIs** |  |  |  |  |  |
| yes | 81 (56.3%) | 84 (57.9%) | 84 (57.9%) | 79 (53.7%) | 328 (56.5%) |
| no | 63 (43.8%) | 61 (42.1%) | 61 (42.1%) | 68 (46.3%) | 253 (43.5%) |

**Exploratory Factor Analyses**

Exploratory factor analyses (EFAs) were conducted using the psych package (Version 2.4.12) in R (4.2.2). Factors were extracted using the minimum residual method (minres). The number of factors was determined based on parallel analysis (Horn, 1965). For the multi-factor solution, an oblique rotation (oblimin) was applied because the factors were expected to correlate. Following common conventions, factor loadings ≥ |.30| were considered substantive (e.g., Tabachnick & Fidell, 2019).

**Table S2**

*Standardized Factor Loadings for the One-Factor Solution of the Expectation Items*

| Item | *MR*1 | *h*² | *u*² |
| --- | --- | --- | --- |
| EXP_1 | 0.92 | 0.84 | 0.16 |
| EXP_2 | 0.66 | 0.43 | 0.57 |
| EXP_3 | 0.64 | 0.41 | 0.59 |
| EXP_4 | 0.76 | 0.58 | 0.42 |
| EXP_5 | 0.89 | 0.80 | 0.20 |
| EXP_6 | 0.86 | 0.74 | 0.26 |
| SS loadings | 3.80 |  |  |
| Proportion of variance | 0.63 |  |  |

*Note*. *N* = 581. Extraction method: minimum residual. *MR*1 = factor loading on Factor 1; *h*² = communality; *u*² = uniqueness. Fit indices: χ²(9) = 720.44, *p* < .001; RMSEA = .369, 90% CI [.347, .392]; TLI = .618; RMSR = .12; BIC = 663.16.

**Table S3**

*Standardized Pattern Matrix for the Two-Factor Solution of Expectation and Intention Items*

| Scale | Item | *MR*1 | *MR*2 | *h*² | *u*² |
| --- | --- | --- | --- | --- | --- |
| *Expectations* | EXP_1 | **0.77** | 0.20 | 0.80 | 0.20 |
|  | EXP_2 | 0.00 | **0.93** | 0.86 | 0.14 |
|  | EXP_3 | 0.04 | **0.84** | 0.74 | 0.26 |
|  | EXP_4 | **0.58** | 0.24 | 0.54 | 0.46 |
|  | EXP_5 | **0.95** | 0.00 | 0.89 | 0.11 |
|  | EXP_6 | **0.96** | −0.04 | 0.89 | 0.11 |
| *Intentions* | v_64 | **0.97** | −0.06 | 0.87 | 0.13 |
|  | v_65 | **0.86** | −0.09 | 0.66 | 0.34 |
|  | v_66 | **0.70** | 0.15 | 0.62 | 0.38 |
| SS loadings |  | 5.03 | 1.84 |  |  |
| Proportion of variance |  | 0.56 | 0.20 |  |  |
| Cumulative variance |  | 0.56 | 0.76 |  |  |

*Note*. *N* = 581. Extraction method: minimum residual; rotation: oblimin. Loadings ≥ |.30| are shown in bold. *MR*1, *MR*2 = pattern coefficients on Factors 1 and 2; *h*² = communality; *u*² = uniqueness. Factor correlation: *r* = .54. Fit indices: χ²(19) = 181.39, *p* < .001; RMSEA = .121, 90% CI [.106, .138]; TLI = .94; RMSR = .02; BIC = 60.46.

**Intentions to take antibiotics**

**Preregistered Hypotheses**

We preregistered two main effects on the intention to take antibiotics:
H1 (individual consequences):
Intentions to use antibiotics to treat the UTI will be lower when the individual consequences of antibiotic use will be communicated (compared to the control group).
H2 (societal consequences):
Intentions to use antibiotics to treat the UTI will be lower when the societal consequences of antibiotic use will be communicated (compared to the control group).

**Measure**

*Intention to take antibiotics* was measured with three items (“I would insist on getting antibiotics to cure my urinary tract infection.”, “I would go to see another doctor to get a prescription for antibiotics.”, “If I get infected with a urinary tract infection, I would take antibiotics.”) on a 7-point Likert scale (Ω= .92).

**Results**

A 2 (individual consequences: communicated vs. not communicated) x 2 (societal consequences: communicated vs. not communicated) between-subjects ANOVA revealed that communicating societal consequences did not lower intentions to take antibiotics, *F*(1, 577) = 3.01, *p* = .084, η²ₚ = .005, but communicating individual consequences did not either, F(1, 577) = 2.04,  = .154, η_p_² = .004. The interaction was not significant, F(1, 577) = 1.60, *p* = .206, η_p_² < .001. Thus, neither H1 nor H2 was supported.

**Table S4**

Equivalence Tests on Trust

| Contrast | Groups compared | *t*(lower), *p* | *t*(upper),  *p* | 90% CI (effect) | NHST *t*, *p* | Decision |
| --- | --- | --- | --- | --- | --- | --- |
| INDIVIDUAL | control vs individual | 1.66, .049 | −3.16, .001 | [−0.211, 0.079] | −0.75, .455 | Equivalent (TOST); NHST n.s. |
| SOCIETAL | no societal vs societal | 0.90, .184 | −3.92, < .001 | [−0.278, 0.012] | −1.51, .131 | Not equivalent (TOST); NHST n.s. |

*Note.* Equivalence testing (TOST, α = .05, SESOI = ±0.20 Cohen’s *d*) on trust used raw-score bounds of −0.212 to 0.212 for both contrasts**.**

**Study 2**

**Table S5**

| *Overview of participant characteristics by individual conditions* | | | |
| --- | --- | --- | --- |
|  | Control (N=378) | Individual (N=387) | Overall (N=765) |
| Age (years) |  |  |  |
| Mean (SD) | 40.8 (14.1) | 40.3 (14.1) | 40.6 (14.1) |
| Median [Min, Max] | 37.5 [18.0, 79.0] | 38.0 [18.0, 80.0] | 38.0 [18.0, 80.0] |
| Age distribution |  |  |  |
| 18-30 | 113 (29.9%) | 127 (32.8%) | 240 (31.4%) |
| 31-40 | 103 (27.2%) | 93 (24.0%) | 196 (25.6%) |
| 41-50 | 55 (14.6%) | 63 (16.3%) | 118 (15.4%) |
| 51-60 | 68 (18.0%) | 65 (16.8%) | 133 (17.4%) |
| 61-70 | 31 (8.2%) | 34 (8.8%) | 65 (8.5%) |
| 71-80 | 8 (2.1%) | 5 (1.3%) | 13 (1.7%) |
| 81-94 | 0 (0%) | 0 (0%) | 0 (0%) |
| Education |  |  |  |
| no school | 0 (0%) | 0 (0%) | 0 (0%) |
| some high school | 6 (1.6%) | 8 (2.1%) | 14 (1.8%) |
| high school diploma or equivalent | 48 (12.7%) | 78 (20.2%) | 126 (16.5%) |
| some college but no degree | 38 (10.1%) | 25 (6.5%) | 63 (8.2%) |
| Trade/technical/vocational training | 31 (8.2%) | 29 (7.5%) | 60 (7.8%) |
| associate’s degree | 7 (1.9%) | 3 (0.8%) | 10 (1.3%) |
| bachelor’s degree | 150 (39.7%) | 141 (36.4%) | 291 (38.0%) |
| master’s degree | 76 (20.1%) | 85 (22.0%) | 161 (21.0%) |
| Professional degree | 10 (2.6%) | 8 (2.1%) | 18 (2.4%) |
| doctorate | 8 (2.1%) | 6 (1.6%) | 14 (1.8%) |
| other | 4 (1.1%) | 4 (1.0%) | 8 (1.0%) |
| Experience with UTIs |  |  |  |
| yes | 234 (61.9%) | 234 (60.5%) | 468 (61.2%) |
| no | 144 (38.1%) | 153 (39.5%) | 297 (38.8%) |

**Intentions to Take Antibiotics**

No hypotheses regarding intentions were pregistered for Study 2.

**Measure**

The intention to take antibiotics was assessed with a single dichotomous item: "Given your doctor just handed you the receipt, would you take the antibiotic?" (yes = 0, no = 1).

**Results**

Because the outcome was binary, we analyzed intentions to take antibiotics using logistic regression with the three message conditions (individual, societal, societal-including-inequalities) and their interactions as predictors, with the control condition as the reference category. Relative to the control condition, all three communication conditions reduced the odds of intending to take antibiotics: individual message, *OR* = 0.31, *p* < .001; societal message, *OR* = 0.30, *p* < .001; societal-including-inequalities message, *OR* = 0.29, *p* < .001. The interactions indicated sub-additive combinations of the messages (individual × societal: *OR* = 3.73, *p* < .001; individual × societal-including-inequalities: *OR* = 2.33, *p* = .023), meaning that the combined effects were smaller than the product of the corresponding main effects. Overall, intentions to take antibiotics were highest in the control condition and lower in all communication conditions.

**Table S6**

Equivalence Tests on Trust

| Contrast | Groups compared | Equivalence bounds (raw) | *t*lower, *p* | *t*upper,  *p* | 90% CI (effect) | NHST *t*,  *p* | Decision |
| --- | --- | --- | --- | --- | --- | --- | --- |
| INDIVIDUAL | control vs individual | −0.306 to 0.306 | 7.65, < .001 | 2.11, .983 | [0.358, 0.722] | 4.88, < .001 | Not equivalent; NHST sig. |
| SOCIETAL (1) | no societal vs societal | −0.488 to 0.488 | 7.38, < .001 | 0.324, .627 | [0.305, 0.760] | 3.85, < .001 | Not equivalent; NHST sig. |
| SOCIETAL (2) | no societal vs societal + inequalities | −0.456 to 0.456 | 8.50, < .001 | 1.70, .955 | [0.462, 0.904] | 5.10, < .001 | Not equivalent; NHST sig. |
| SOCIETAL (3) | societal vs societal + inequalities | −0.456 to 0.456 | 4.54, < .001 | −2.28, .011 | [−0.069, 0.370] | 1.13, .260 | Equivalent; NHST ns |

*Note.* Two one-sided tests (TOST) conducted with SESOI = ±0.20 Cohen’s *d*, α = .05. CI = confidence interval; NHST = null-hypothesis significance test; sig. = significant; ns = non-significant. Equivalence requires both one-sided tests to be significant (*p* < .05).

**Table S7**

Logistic Regression Predicting Antibiotic Intake

| Predictor | OR | 95% CI | p |
| --- | --- | --- | --- |
| Intercept | 2.472 | [1.679, 3.641] | < .001 |
| INDIVIDUAL (individual vs control) | 0.310 | [0.184, 0.522] | < .001 |
| SOCIETAL: societal consequences (vs none) | 0.299 | [0.177, 0.505] | < .001 |
| SOCIETAL: societal + inequalities (vs none) | 0.294 | [0.174, 0.496] | < .001 |
| INDIVIDUAL × SOCIETAL: societal consequences | 3.734 | [1.824, 7.647] | < .001 |
| INDIVIDUAL × SOCIETAL: societal + inequalities | 2.325 | [1.123, 4.813] | .023 |

*Note.* Logistic regression with Wald 95% confidence intervals. OR < 1 indicates lower odds of intending to take antibiotics; OR > 1 indicates higher odds.

**Table S8**

Logistic Regression Predicting Intake of Plant-based Alternative

| Predictor | OR | 95% CI | p |
| --- | --- | --- | --- |
| Intercept | 1.016 | [0.716, 1.443] | .929 |
| INDIVIDUAL (individual vs control) | 2.357 | [1.407, 3.948] | .001 |
| SOCIETAL: societal consequences (vs none) | 2.488 | [1.477, 4.190] | .001 |
| SOCIETAL: societal + inequalities (vs none) | 2.663 | [1.572, 4.510] | < .001 |
| INDIVIDUAL × SOCIETAL: societal consequences | 0.269 | [0.129, 0.560] | < .001 |
| INDIVIDUAL × SOCIETAL: societal + inequalities | 0.535 | [0.248, 1.156] | .112 |

*Note.* Logistic regression with Wald 95% confidence intervals. OR < 1 indicates lower odds of intending to take plant-based alternative; OR > 1 indicates higher odds.

**Study 3**

**Table S9**

| *Overview of participant characteristics by recommendation conditions* | | | | |
| --- | --- | --- | --- | --- |
|  | **control (N=569)** | **individual (N=570)** | **societal (N=572)** | **Overall (N=1711)** |
| **Age mean** |  |  |  |  |
| Mean (SD) | 43.0 (13.3) | 42.0 (13.3) | 42.2 (13.7) | 42.4 (13.4) |
| Median [Min, Max] | 42.0 [19.0, 81.0] | 40.0 [19.0, 84.0] | 40.5 [19.0, 79.0] | 41.0 [19.0, 84.0] |
| **Age distribution** |  |  |  |  |
| 18-30 | 116 (20.4%) | 126 (22.1%) | 138 (24.1%) | 380 (22.2%) |
| 31-40 | 148 (26.0%) | 167 (29.3%) | 148 (25.9%) | 463 (27.1%) |
| 41-50 | 125 (22.0%) | 107 (18.8%) | 114 (19.9%) | 346 (20.2%) |
| 51-60 | 117 (20.6%) | 115 (20.2%) | 103 (18.0%) | 335 (19.6%) |
| 61-70 | 54 (9.5%) | 44 (7.7%) | 57 (10.0%) | 155 (9.1%) |
| 71-80 | 8 (1.4%) | 10 (1.8%) | 12 (2.1%) | 30 (1.8%) |
| 81-94 | 1 (0.2%) | 1 (0.2%) | 0 (0%) | 2 (0.1%) |
| **Education** |  |  |  |  |
| no school | 0 (0%) | 0 (0%) | 1 (0.2%) | 1 (0.1%) |
| some high school | 9 (1.6%) | 11 (1.9%) | 8 (1.4%) | 28 (1.6%) |
| high school diploma or equivalent | 78 (13.7%) | 64 (11.2%) | 76 (13.3%) | 218 (12.7%) |
| some college but no degree | 43 (7.6%) | 64 (11.2%) | 63 (11.0%) | 170 (9.9%) |
| Trade/technical/vocational training | 55 (9.7%) | 55 (9.6%) | 40 (7.0%) | 150 (8.8%) |
| associate's degree | 10 (1.8%) | 11 (1.9%) | 10 (1.7%) | 31 (1.8%) |
| bachelor's degree | 228 (40.1%) | 239 (41.9%) | 243 (42.5%) | 710 (41.5%) |
| master's degree | 100 (17.6%) | 88 (15.4%) | 96 (16.8%) | 284 (16.6%) |
| Professional degree | 17 (3.0%) | 15 (2.6%) | 18 (3.1%) | 50 (2.9%) |
| doctorate | 23 (4.0%) | 23 (4.0%) | 17 (3.0%) | 63 (3.7%) |
| other | 6 (1.1%) | 0 (0%) | 0 (0%) | 6 (0.4%) |
| **Experience with UTIs** |  |  |  |  |
| yes | 284 (49.9%) | 280 (49.1%) | 287 (50.2%) | 851 (49.7%) |
| no | 285 (50.1%) | 290 (50.9%) | 285 (49.8%) | 860 (50.3%) |

**Intentions to Take Antibiotics**

No hypotheses regarding intentions were preregistered in Study 3.

**Measure**

Intentions were assessed with four dichotomous items (1 = *yes*, 0 = *no*): two items regarding antibiotics (intention to buy antibiotics and intention to take them immediately upon getting home) and two items regarding a plant-based alternative (intention to buy the alternative and intention to take it immediately upon getting home).

**Results**

For antibiotics, 63% of the participants reported that they planned to buy them, but only 34% indicated that they would take them immediately upon arriving home. Notably, 29% of participants intended to buy antibiotics without taking them right away, suggesting that a substantial proportion of participants treated purchasing antibiotics as a precautionary measure rather than as an immediate intention to use them. For the plant-based alternative, 66% of the participants reported intending to buy it and 62% reported intending to take it immediately. Only 4% of the participants indicated buying the plant-based option without taking it, suggesting that participants were much less likely to purchase the plant-based alternative as a precautionary stock. We further examined this buy–take pattern using logistic regression (see Tables S8–S11). Societal messaging significantly increased the selection of the plant-based alternative, whereas no significant effects of the experimental conditions emerged for any of the antibiotic-related intentions.

**Table S10**

Logistic Regression Predicting Buying Antibiotics (no = 0, yes = 1): Odds Ratios (Wald 95% CI)

| Predictor | Model 1 OR | 95% CI | p | Stepwise OR | 95% CI | p |
| --- | --- | --- | --- | --- | --- | --- |
| Age | 0.99 | [0.98, 0.99] | .001 | 0.99 | [0.98, 0.99] | < .001 |
| Education (middle) | 1.18 | [0.84, 1.66] | .332 | — | — | — |
| Education (high) | 1.19 | [0.89, 1.59] | .235 | — | — | — |
| UTI severity | 1.22 | [1.11, 1.35] | < .001 | 1.23 | [1.11, 1.35] | < .001 |
| No-consequence × ABX | 2.22 | [1.58, 3.14] | < .001 | 2.21 | [1.57, 3.12] | < .001 |
| Individual-consequence × ABX | 2.31 | [1.64, 3.25] | < .001 | 2.31 | [1.65, 3.26] | < .001 |
| Societal-consequence × ABX | 1.66 | [1.19, 2.34] | .003 | 1.65 | [1.18, 2.32] | .004 |
| No-consequence × ABX+Alt | 1.85 | [1.32, 2.60] | < .001 | 1.85 | [1.32, 2.59] | < .001 |
| Individual-consequence × ABX+Alt | 1.26 | [0.90, 1.75] | .181 | 1.25 | [0.90, 1.75] | .186 |
| Tjur’s R² | .040 |  |  | .039 |  |  |

*Note.* OR > 1 indicates higher odds of buying.

**Table S11**

Logistic Regression Predicting Taking Antibiotics (no = 0, yes = 1): Odds Ratios (Wald 95% CI)

| Predictor | Model 1 OR | 95% CI | p | Stepwise OR | 95% CI | p |
| --- | --- | --- | --- | --- | --- | --- |
| Age | 0.99 | [0.98, 0.99] | .001 | 0.99 | [0.98, 0.99] | < .001 |
| Education (middle) | 1.17 | [0.83, 1.65] | .365 | 1.17 | [0.83, 1.65] | .373 |
| Education (high) | 0.85 | [0.63, 1.14] | .264 | 0.85 | [0.64, 1.15] | .283 |
| UTI severity | 1.07 | [0.97, 1.18] | .168 | — | — | — |
| No-consequence × ABX | 1.84 | [1.29, 2.62] | .001 | 1.85 | [1.30, 2.64] | .001 |
| Individual-consequence × ABX | 1.74 | [1.23, 2.48] | .002 | 1.73 | [1.22, 2.47] | .002 |
| Societal-consequence × ABX | 1.31 | [0.91, 1.89] | .152 | 1.32 | [0.92, 1.91] | .132 |
| No-consequence × ABX+Alt | 1.91 | [1.34, 2.73] | < .001 | 1.91 | [1.34, 2.73] | < .001 |
| Individual-consequence × ABX+Alt | 1.43 | [0.99, 2.06] | .054 | 1.43 | [0.99, 2.05] | .055 |
| Tjur’s R² | .022 |  |  | .021 |  |  |

*Note.* OR > 1 indicates higher odds of taking.

**Table S12**

Logistic Regression Predicting Buying Plant-Based Alternative (no = 0, yes = 1): Odds Ratios (Wald 95% CI)

| Predictor | Model 1 OR | 95% CI | p | Stepwise OR | 95% CI | p |
| --- | --- | --- | --- | --- | --- | --- |
| Age | 1.01 | [0.99, 1.02] | .366 | — | — | — |
| Education (middle) | 0.86 | [0.53, 1.39] | .543 | — | — | — |
| Education (high) | 1.16 | [0.77, 1.75] | .471 | — | — | — |
| UTI severity | 1.06 | [0.93, 1.21] | .351 | — | — | — |
| Individual-consequence (vs none) | 1.10 | [0.78, 1.56] | .582 | 1.09 | [0.77, 1.53] | .641 |
| Societal-consequence (vs none) | 1.59 | [1.12, 2.26] | .009 | 1.59 | [1.12, 2.26] | .009 |
| Tjur’s R² | .014 |  |  | .009 |  |  |

*Note.* OR > 1 indicates higher odds of buying the plant-based alternative.

**Table S13**

Logistic Regression Predicting Taking Plant-Based Alternative (no = 0, yes = 1): Odds Ratios (Wald 95% CI)

| Predictor | Model 1 OR | 95% CI | p | Stepwise OR | 95% CI | p |
| --- | --- | --- | --- | --- | --- | --- |
| Age | 1.00 | [0.99, 1.01] | .564 | — | — | — |
| Education (middle) | 0.95 | [0.59, 1.52] | .820 | — | — | — |
| Education (high) | 1.13 | [0.75, 1.69] | .554 | — | — | — |
| UTI severity | 1.03 | [0.91, 1.18] | .619 | — | — | — |
| Individual-consequence (vs none) | 1.10 | [0.78, 1.54] | .582 | 1.09 | [0.78, 1.53] | .617 |
| Societal-consequence (vs none) | 1.80 | [1.28, 2.54] | .001 | 1.80 | [1.28, 2.54] | .001 |
| Tjur’s R² | .017 |  |  | .015 |  |  |

*Note.* OR > 1 indicates higher odds of taking the plant-based alternative.
